# Using a stable protein scaffold to display peptides that bind to alpha‐synuclein fibrils

**DOI:** 10.1002/pro.70150

**Published:** 2025-05-15

**Authors:** Samuel Bismut, Matthias M. Schneider, Masashi Miyasaki, Yuqing Feng, Ellis J. Wilde, M. Dylan Gunawardena, Tuomas P. J. Knowles, Gabrielle S. Kaminski Schierle, Laura S. Itzhaki, Janet R. Kumita

**Affiliations:** ^1^ Department of Pharmacology University of Cambridge Cambridge UK; ^2^ Yusuf Hamied Department of Chemistry University of Cambridge Cambridge UK; ^3^ Department of Chemical Engineering and Biotechnology University of Cambridge Cambridge UK

**Keywords:** amyloid fibrils, fibril‐binders, protein scaffolds, α‐synuclein

## Abstract

Amyloid fibrils are ordered aggregates that are a pathological hallmark of many neurodegenerative disorders including Alzheimer's disease and Parkinson's disease. The process of amyloid formation involves a complex cascade by which soluble monomeric protein converts to insoluble, ordered aggregates (amyloid fibrils). Although inhibiting the aggregation pathway is a key target for therapeutic development, the heterogeneous collection of aggregation‐prone species formed in this process, including oligomers, protofibrils, and fibrils, represents other targets for modifying disease pathology. Developing molecules that can bind to amyloid fibrils and potentially disrupt the harmful interactions between the fibrils and the cellular components would be advantageous. Designing peptide modulators for α‐synuclein aggregation is of great interest; however, effective inhibitory peptides are often hydrophobic and hence difficult to handle. Therefore, developing strategies to display these peptides in a soluble scaffold would be very beneficial. Here we demonstrate that the ultra‐stable consensus‐designed tetratricopeptide repeat (CTPR) protein scaffold can be grafted with “KLVFF” derived peptides previously identified to inhibit protein aggregation and interact with amyloid fibrils to produce proteins that bind along the surface of α‐synuclein fibrils with micromolar affinity. Given the ability to insert hydrophobic peptides to produce soluble, CTPR‐based binders, this method may prove beneficial in screening for peptide modulators of protein aggregation.

## 1 INTRODUCTION

Protein misfolding and aggregation into amyloid fibrils has been identified as a pathological hallmark for several neurodegenerative disorders, including Alzheimer's disease (AD) and Parkinson's disease (PD) (Chiti & Dobson, 2017). In PD, the 14 kDa protein α‐synuclein aggregates to form cross‐β amyloid fibrils via a pathway that populates less organized oligomeric intermediates (Li et al., 2001; Serpell et al., 2000). Although it is believed that these oligomeric species are the key toxic species in the context of disease (Cremades et al., 2012; Fusco et al., 2017; Glabe, 2006), the mature fibrils have a key role in propagating the aggregation process, for example, through secondary nucleation processes where protofibrils can accelerate amyloid formation through their fibril ends or fibril surfaces (Buell et al., 2014; Cohen et al., 2013). Along with significant efforts to identify inhibitors of the aggregation process of α‐synuclein (Agerschou et al., 2019; Cheruvara et al., 2015; Chia et al., 2023; Meade et al., 2020; Santos et al., 2021), there has been great interest in identifying molecules that can bind specifically to the α‐synuclein fibrils in order to modulate the aggregation process (Monsellier et al., 2020; Sangwan et al., 2020; Scheidt et al., 2021; Wallace et al., 2024). Furthermore, fibril‐binding strategies are important for targeted protein degradation approaches to direct aggregates to the cell's natural proteostasis machinery for clearance (Lee et al., 2023; Tomoshige & Ishikawa, 2021). Recent advances in de novo design have enabled the development of peptides and mini‐proteins that bind to amyloid fibrils or with regions of monomeric amyloid‐related proteins, creating high‐affinity scaffolds that can modulate aggregation pathways (Agerschou et al., 2019; Sahtoe et al., 2024; Sangwan et al., 2020; Wallace et al., 2024). Moreover, rational antibody design strategies have made it possible to develop grafted amyloid‐motif antibodies (gammabodies) that bind specifically to Aβ fibrils with high affinity (Julian et al., 2019; Lee et al., 2016) and to create other conformational‐specific antibodies that can recognize Aβ_1‐42_ and α‐synuclein oligomers (Aprile et al., 2020; Kulenkampff et al., 2022). Interestingly, peptides derived from the KLVFF peptide (amino acids 16–20; Aβ_1‐42_) that known to modulate Aβ fibril formation (Tjernberg et al., 1996) have also been reported to bind to Aβ fibrils and α‐synuclein fibrils (Aoraha et al., 2015; Wood et al., 2020). There are several studies that use the KLVFFAE peptide to bind to Aβ fibrils, but the peptide itself is aggregation‐prone and consequently often requires methods to increase its solubility and efficacy, including terminal‐capping strategies (Aoraha et al., 2015), incorporating D‐amino acids (Horsley et al., 2020), and coupling to gold and gadolinium nanoparticles (Gao et al., 2015; Plissonneau et al., 2016).

To test potential fibril‐binding peptides and probe the mechanism by which they interact with amyloid fibrils, having an ultra‐stable scaffold for their display would be advantageous. The consensus‐designed tetratricopeptide repeat protein (CTPR) scaffold comprises tandem arrays of a 34‐residue α‐helix‐turn‐α‐helix motif, and their simple, modular architecture and high stability make them useful tools for protein engineering (Main et al., 2003; Perez‐Riba & Itzhaki, 2019). A rational design approach can be used to endow CTPR scaffolds with additional functionality including specific client recruitment via grafting of single and multiple copies of short linear binding motifs (SLiMs) between adjacent repeats in CTPR scaffolds (ranging from 2 to 6 repeat units), with diverse, precise, and predictable geometries for multivalent and multi‐functional display (Diamante et al., 2021; Madden et al., 2019; Ng et al., 2024; Perez‐Riba & Itzhaki, 2019). Here we demonstrate that the KLVFFAE peptide and a variation, KLVFWAK (Wood et al., 2020), can be grafted onto our 3‐repeat CTPR scaffold to produce soluble chimeras (FibSyn‐CTPR and FibSyn2‐CTPR) that can bind with micromolar affinity to α‐synuclein fibrils, along with a 4‐repeat version containing two KLVFFAE motifs (between repeat 1 and 2 and between repeat 3 and 4; 2XFibSyn‐CTPR4) that displays similar affinity for α‐synuclein fibrils with an increase in the number of associated molecules to the fibrils. Incubation of fluorophore‐labeled fibril‐binding CTPRs with unlabeled α‐synuclein fibrils resulted in full visualization of the fibrillar structures by fluorescence microscopy, revealing that the interaction is with the entire surface of the fibrils and not isolated to specific regions. Finally, constraining these peptides in the inter‐repeat loop of the CTPR scaffold imparts a preference of the FibSyn‐CTPR variants for α‐synuclein fibrils as compared to pre‐formed Aβ_1‐42_ fibrils.

## 2 RESULTS

**FIGURE 1:**
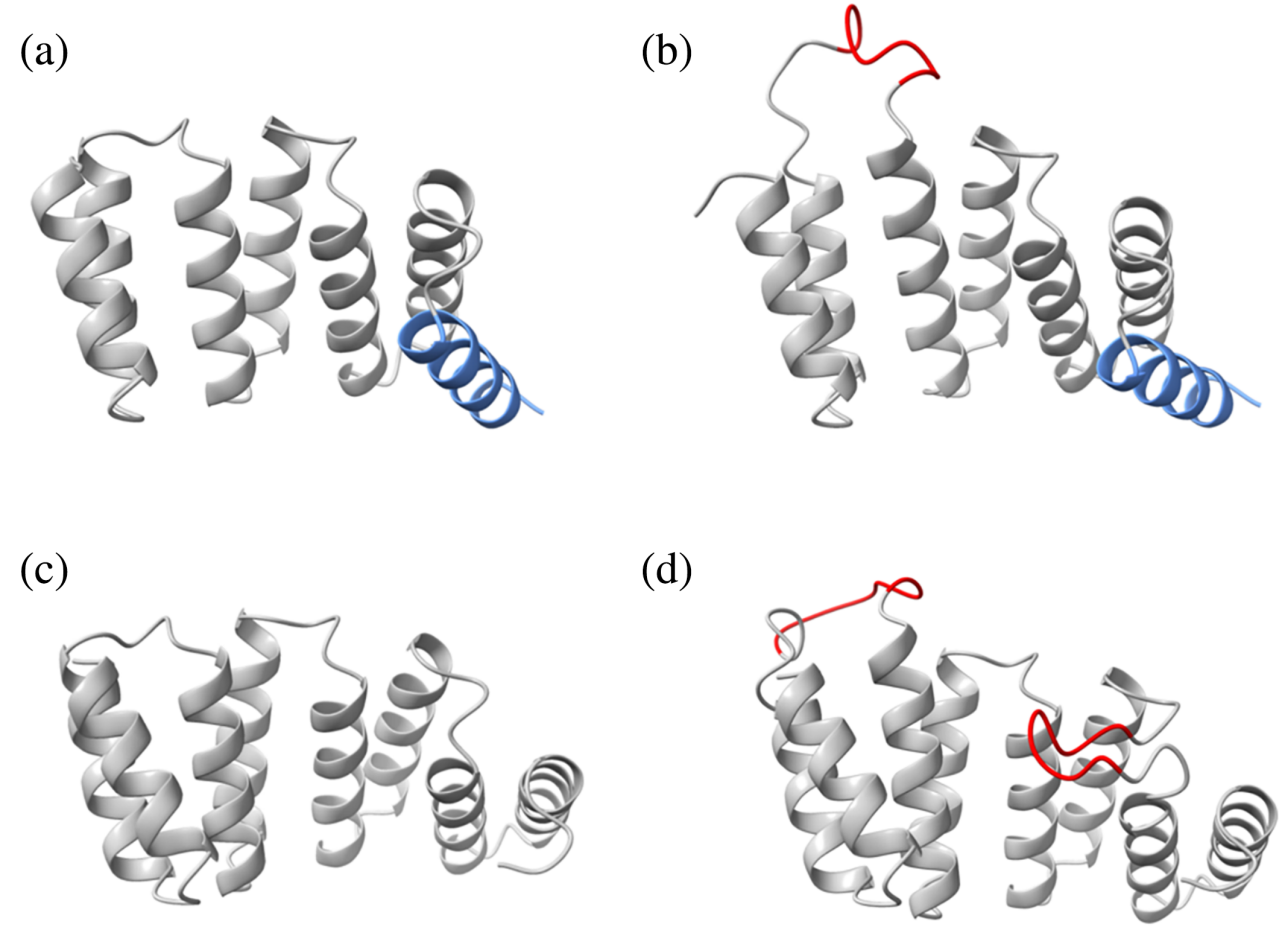
Schematic diagrams of CTPR scaffolds with incorporated fibril‐binding motifs. Alphafold2 models of (a) the CTPR3 scaffold protein containing three‐repeat units and a solvating helix (blue), (b) FibSyn‐CTPR3 containing three‐repeat units, a solvating helix and a fibril‐binding motif between repeat 1–2 (red), (c) CTPR4 containing four‐repeat units and D) 2XFibSyn‐CTPR4 consisting of four‐repeat units with a fibril‐binding motif between repeat 1–2 and between repeat 3–4 (red). The peptide sequence for FibSyn is KLVFFAE and for FibSyn2 it is KLVFWAK. Full protein sequences are available in Table S1.

Single peptide motifs (KLVFFAE or KLVFWAK) were grafted between repeat 1–2 of a CTPR3 scaffold designed with DPRS inter‐repeat loops and with a solvating helix (Main et al., 2003) to create FibSyn‐CTPR3 and FibSyn2‐CTPR3, respectively. A CTPR4 scaffold containing the KLVFFAE peptide between repeat 1–2 and between repeat 3–4 (2XFibSyn‐CTPR4) was also designed (Figure 1). The CTPR4 design has DPNN inter‐repeat loops and does not include a solvating helix, as Diamante and co‐workers showed that the CTPR4 scaffold can accommodate 2‐grafted binding loops without compromising the solubility or protein yield (Diamante et al., 2021). In all cases, DPNN linker regions flanked the fibril‐binding peptides to increase the flexibility of the loops to maximize binding and to preserve the chemical environment of the residues adjacent to the loops (Madden et al., 2019).

### 2.1 Fibril‐binding CTPRs retain scaffold structure and high stability

**FIGURE 2:**
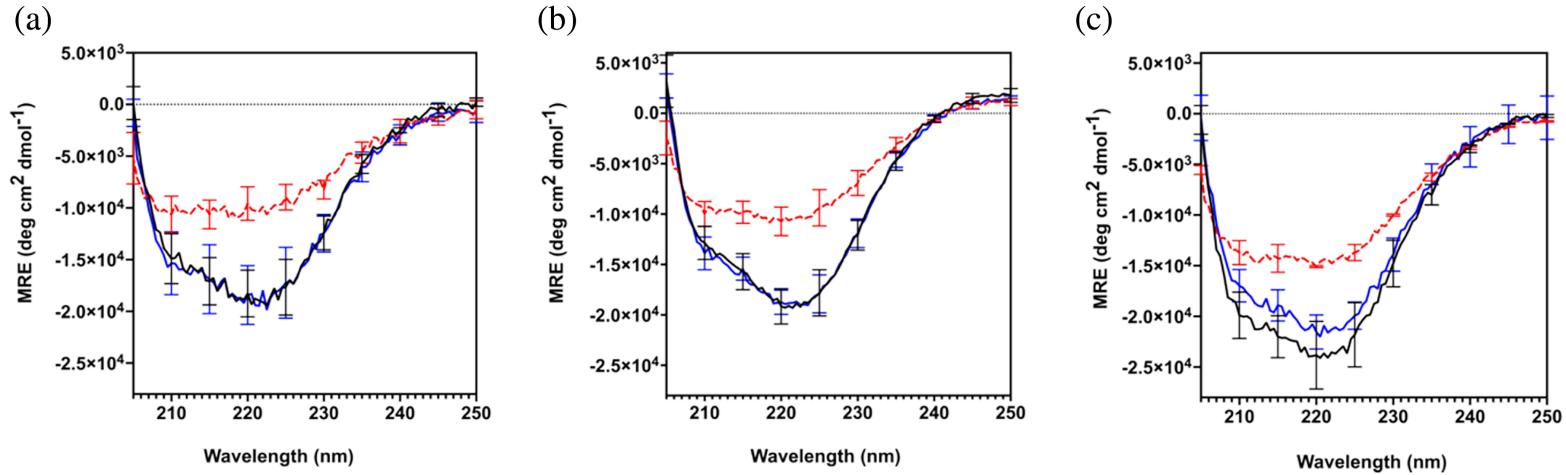
CD analysis of CTPR variant secondary structure. Far‐UV CD spectra of (a) FibSyn‐CTPR3, (b) FibSyn2‐CTPR3 and (c) 2XFibSyn‐CTPR4 at 20°C (black), 90°C (red) and returning to 20°C (blue). Protein concentration was 5 μM in 50 mM sodium phosphate pH 7.5, 150 mM NaCl. Error bars are standard deviation calculated from three independent experiments.

All CTPR variants (and their respective negative controls wild‐type (WT) CTPR3 and CTPR4, containing no additional inter‐repeat loops) were expressed and purified, resulting in protein concentrations in the range of 120–320 μM. We note that the 2XFibSyn CTPR4 has consistently lower concentrations after purification and evidence of aggregation has been observed above 100 μM (upon thawing after storage). The insertion of the fibril‐binding loops does not alter the native fold of the CTPR variants compared with their WT controls, as measured by circular dichroism (CD) spectroscopy (Figures 2 and S1), and as expected, the inserted loops decrease the native‐state stability as determined by GdnHCl denaturation assays, but only by a modest amount (Table 1 and Figures S1 and S2). In agreement with the chemical denaturation, thermal unfolding, assayed by measuring the CD spectrum at 90°C, shows that the helical content of the CTPR variants decreases more than that of the WT controls (Figure S2) but the helicity is recovered when returning to 20°C (Figure 2).

**TABLE 1:** Parameters obtained from two‐state fit of the GdnHCl‐induced chemical denaturation experiments for CTPR variants.

Protein	*D*_50%_ (M)	*m*‐value (kcal mol^−1^ M^−1^)
CTPR3	3.6 ± 0.1	2.6 ± 0.2
FibSyn‐CTPR3	3.4 ± 0.1	2.1 ± 0.5
FibSyn2‐CTPR3	2.8 ± 0.2	1.3 ± 0.7
CTPR4	4.8 ± 0.1	3.8 ± 0.6
2XFibSyn‐CTPR4	3.8 ± 0.1	1.4 ± 0.5

### 2.2 Fibril‐binding CTPR variants interact with α‐synuclein fibrils

**FIGURE 3:**
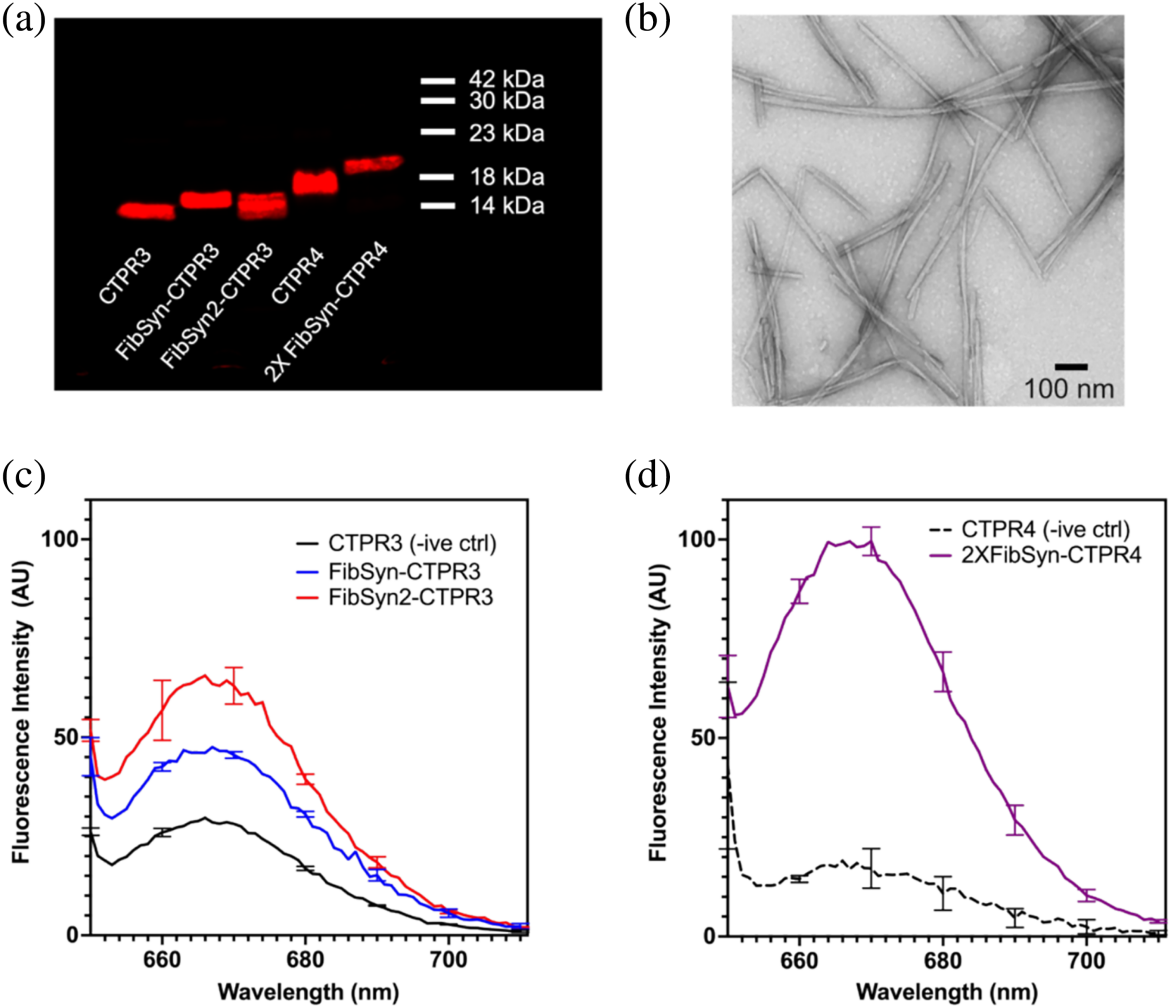
Fibril pulldown assay to assess interactions between CTPR variants and α‐synuclein fibrils. (a) SDS‐PAGE analysis of Alexa Fluor™ 647 labeled CTPR variants. (b) TEM image of α‐synuclein fibrils prior to incubation with fluorescently labeled CTPRs. (c) Fluorescence emission spectrum for CTPR3 (negative control), FibSyn‐CTPR3 and FibSyn2‐CTPR3 bound α‐synuclein fibrils. (d) Fluorescence emission spectrum for CTPR4 (negative control) and 2XFibSyn‐CTPR4 bound α‐synuclein fibrils. Error bars represent the standard deviation of two independent experiments.

To test whether the CTPR variants with inserted peptide motifs could recognize monomeric or fibrillar α‐synuclein, an initial dot blot assay was performed. After applying monomeric and fibrillar α‐synuclein to nitrocellulose membranes, fluorescently labeled CTPR variants were incubated to look for interactions. FibSyn‐CTPR3, FibSyn2‐CTPR3, and 2XFibSyn‐CTPR4 showed positive interactions with α‐synuclein fibrils but not monomeric α‐synuclein (Figure S3) and CTPR3‐WT showed little interaction. To ensure that these specific interactions were not an artifact of immobilizing the fibrils onto the nitrocellulose membrane, a fibril‐pulldown assay was carried out. CTPR variants containing a single cysteine were labeled with Alexa Fluor™ 647 maleimide (Figure 3a) and incubated with α‐synuclein fibrils (Figure 3b). After extensive washing of the fibril/CTPR complexes to remove non‐specific fluorescently labeled CTPRs, the fluorescence emission spectra were measured (Figure 3c,d) and corrected for labeling efficiency. Using these experiments, we could observe clear binding between the FibSyn‐CTPR3, FibSyn2‐CTPR3, and 2XFibSyn‐CTPR4 variants and α‐synuclein fibrils. We observed lower fluorescence intensity for CTPR3‐WT (Figure 3c) indicative of weaker binding, which we also noted in the dot‐blot assay, whereas CTPR4‐WT showed less fluorescence emission.

### 2.3 Fibril‐binding CTPR variants bind with micromolar affinity and along the entire fibril surface

To measure the binding affinities and stoichiometries of the interactions between the fibril‐binding CTPRs and α‐synuclein fibrils, we applied a microfluidic diffusional sizing (MDS) approach. MDS allows us to determine the hydrodynamic radii (*R*_H_) of individual components in complex mixtures of species and to quantify their relative concentrations (Arosio et al., 2016; Scheidt et al., 2019, 2021). Using fluorescently labeled CTPR variants, we can monitor the change in R_H_ as they bind to large, pre‐formed fibrils (PFFs) of α‐synuclein to quantify these interactions. MDS also provides insights into the stoichiometry of the CTPR variants in relation to the α‐synuclein units.

**FIGURE 4:**
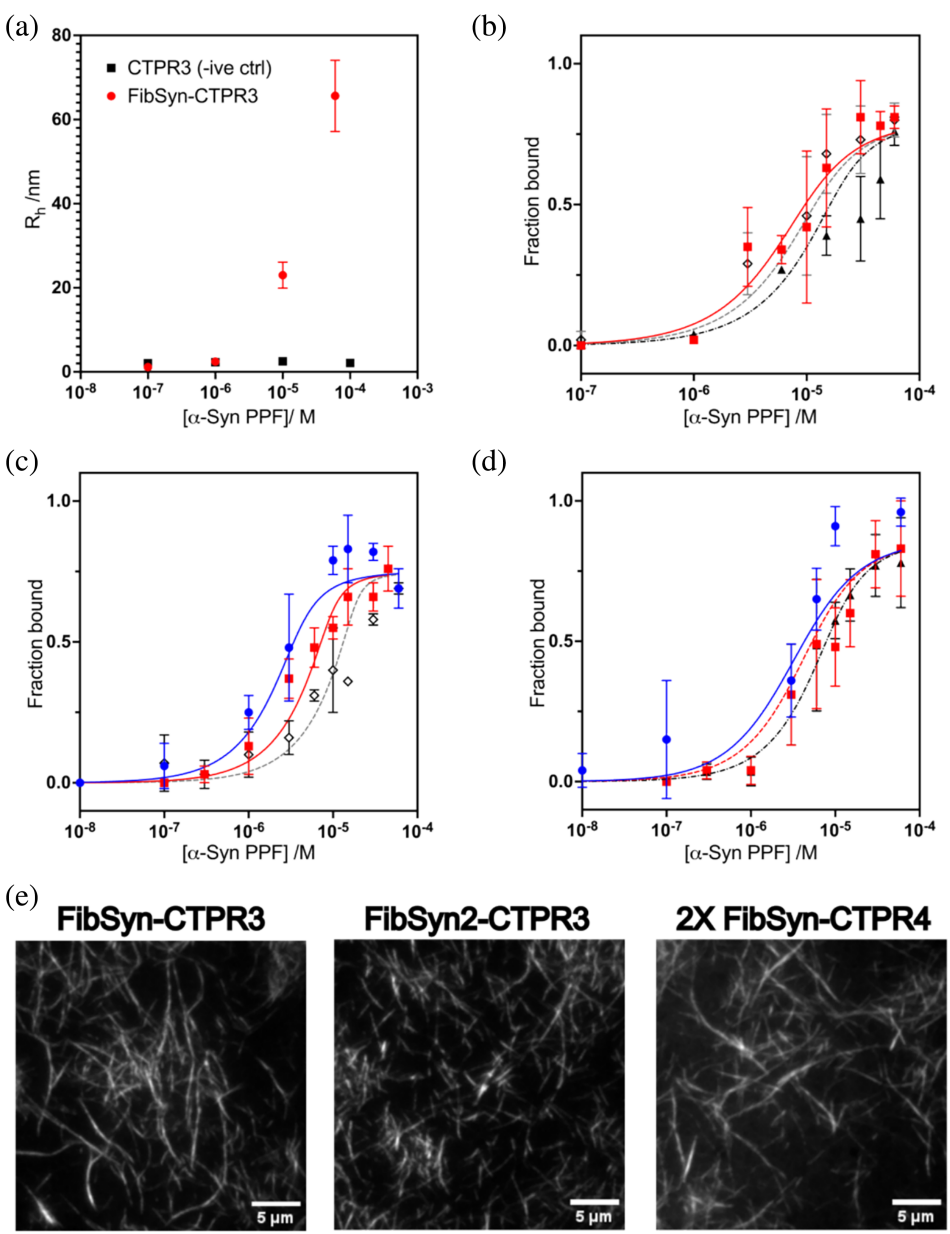
Microfluidic diffusional sizing (MDS) assay to determine binding affinity between fluorescently labeled CTPR variants and α‐synuclein preformed fibrils (PFFs). (a) Initial hydrodynamic radius measurements to compare CTPR3 (negative control) and FibSyn‐CTPR3 interactions with increasing α‐synuclein PFF concentrations. The hydrodynamic radius for FibSyn‐CTPR3 significantly increases in the presence of α‐synuclein PFFs whereas no change is observed for CTPR3 (negative control). Fraction of FibSyn bound to α‐synuclein PFFs at varying concentrations of PFFs for (b) FibSyn‐CTPR3, (c) FibSyn2‐CTPR3 and (d) 2XFibSyn‐CTPR4. Each MDS assay was performed with different concentrations of CTPR variants including 75 nM (blue circles), 200 nM (red squares), 400 nM (black open diamonds) and 500 nM (black triangles). Dissociation constants are shown in Table 2. (e) TIRF microscopy imaging of Alexa Fluor™ 647‐labeled CTPR variants bound to α‐synuclein fibrils (prepared in a similar way to Figure 3 pulldown assay samples). Scale bars represent 5 μm.

An initial comparison of the hydrodynamic radius of Alexa Fluor™ 647‐labeled FibSyn‐CTPR3 with increasing concentrations of unlabeled α‐synuclein PFFs shows a significant increase in R_H_, whereas the same titration with Alexa Fluor™ 647‐labeled CTPR3‐WT shows no binding (Figure 4a). Next, we performed titrations for each fibril‐binding CTPR variant, and by analyzing the fraction of bound CTPR variant at varying PFF concentrations, we determined the dissociation constants and stoichiometries (Table 2). All three fibril‐binding variants had dissociation constants in the low micromolar range. When comparing the stoichiometry, that is, the number of CTPR binders per α‐synuclein unit, the FibSyn‐CTPR3 and FibSyn2‐CTPR3 were very similar, 1 binder per 34 and 1 binder per 45, respectively, whereas for 2XFibSyn‐CTPR4 the stoichiometry was approximately doubled with 1 binder per 14 α‐synuclein units. This result suggests that, although the presence of two KLVFFAE peptide motifs does not increase the affinity of the CTPR variant for the fibrils, both binding motifs interact with the fibrils. In the 2XFibSyn CTPR4, the FibSyn motifs are offset by approximately 90° in the CTPR4 scaffold (Figure 1d) due to the CTPR proteins adopting a super‐helical conformation, with eight repeats completing the superhelical turn (Kajander et al., 2007). This may compromise the individual binding affinity of each site, but as both interact with the fibrils, the combined effect still results in micromolar affinity. Finally, to determine the localization of the fibril‐binding CTPR variants on α‐synuclein fibrils, we imaged the Alexa Fluor™ 647‐labeled CTPR variants bound to α‐synuclein fibrils using total internal reflection fluorescence (TIRF) microscopy (Figure 4e). In all cases, the CTPR variants are distributed throughout the fibril structures.

**TABLE 2:** Binding of CTPR variants with α‐synuclein PFFs.

CTPR variant	Dissociation constant *K*_d_ (μM)	Binding stoichiometry
FibSyn‐CTPR3	3.23 [0.87, 6.90]	1 binder per 34 [4, 66] α‐syn units
FibSyn2‐CTPR3	0.58 [0.02, 1.69]	1 binder per 45 [27, 62] α‐syn units
2XFibSyn‐CTPR4	2.65 [1.47, 4.26]	1 binder per 14 [2, 28] α‐syn units

*Note*: Values calculated from MDS experiments with the upper and lower boundaries of the 95% confidence interval shown in square brackets.

As the KLVFFAE peptide motif is well studied for its interactions with amyloid‐β (Aβ) aggregates (Aoraha et al., 2015; Liu et al., 2023; Plissonneau et al., 2016; Wood et al., 2020), we tested if our fibril‐binding CTPR variants showed specific interactions with pre‐formed Aβ_1‐42_ fibrils, using first a fibril pulldown assay for FibSyn2‐CTPR3 versus CTPR3‐WT and followed by MDS experiments for all three potential binders. In both experiments, we observe no interactions between the fibril‐binding CTPR variants and Aβ_1‐42_ fibrils (Figure S4). It appears that constraining this peptide‐motif within the inter‐repeat loops of the CTPR scaffold imparts a conformational restraint that allows these variants to have a specificity for α‐synuclein fibrils over Aβ_1‐42_ fibrils.

## 3 DISCUSSION

Here we show the utility of the CTPR scaffold to not only display hydrophobic peptides in the inter‐repeat loops to produce highly soluble protein, but to also produce CTPR‐binders that have micromolar affinity for α‐synuclein fibrils. In addition, these CTPR‐binders can be used to label the surfaces of in vitro α‐synuclein to enable visualization of the structures using fluorescence microscopy. It is interesting to note that constraining these peptides in the inter‐repeat loops of the CTPRs introduces a selectivity for α‐synuclein fibrils over Aβ_1‐42_ fibrils that has not been reported for the peptide motifs alone (Aoraha et al., 2015; Wood et al., 2020). Despite the similarities in the cross‐β structure of amyloid fibrils, the physicochemical attributes can differ significantly, and this may impact the fibril‐binding properties of our CTPR‐binders. For example, spectrally resolved point accumulation for imaging in nanoscale topography (sPAINT) microscopy techniques have demonstrated that the surfaces of in vitro α‐synuclein fibrils are more hydrophobic than Aβ fibrils (Bongiovanni et al., 2016). However, further studies are needed to elucidate the binding epitopes on the α‐synuclein fibrils that are recognized by these CTPR‐binders.

Although the interaction of the FibSyn‐CTPRs with the surfaces of the synuclein fibrils may have implications for modulating aggregation kinetics (Buell et al., 2014), attempts to establish the effect on surface‐catalyzed nucleation assays have been inconclusive, in large part due to the kinetic reaction conditions being carried out at a pH that is not ideal for the FibSyn‐CTPRs, following close to the isoelectric points. Within the CTPR scaffold, only 8 residues are essential to ensure correct folding and, therefore, this system is amenable to protein engineering to improve its physicochemical properties (Main et al., 2003; Uribe et al., 2021); rational design approaches may lead to future variants that can be used to modulate aggregation kinetics.

Given the number of peptides reported in the literature that alter α‐synuclein aggregation or bind to different α‐synuclein conformers (Cheruvara et al., 2015; Meade et al., 2020, 2021; Monsellier et al., 2020; Nahomi et al., 2015; Santos et al., 2021; Wood et al., 2020), our CTPR scaffold may provide a facile method to further study their mechanisms of action for modulating amyloid fibril formation and lead to the development of diagnostic tools and potential therapeutics.

## 4 MATERIALS AND METHODS

### 4.1 CTPR plasmid preparation and protein purification

CTPR constructs were cloned using gBlock oligos (Integrated DNA Technologies) into a modified pRSET‐B vector with FastDigest BamHI and HindIII restriction enzymes (ThermoFisher Scientific (UK) Ltd.) and Anza T4 DNA ligase master mix (Invitrogen, Paisley, UK). Plasmids were transformed into chemically competent C41 *E. coli* cells using standard heat‐shock protocols. Colonies were grown at 37°C in 2xYT media (Formedium, Swaffham, UK) containing ampicillin (50 μg/mL). Upon reaching OD_600nm_ of 0.6–0.8, bacterial cultures were induced with isopropyl D‐thiogalactopyranoside (IPTG, 0.5 mM, PanReac AppliChem, Darmstadt, Germany) and incubated (18 h, 20°C, 200 rpm). Cell pellets were collected by centrifugation from 125 mL of culture and resuspended in 15 mL lysis buffer (50 mM sodium phosphate (pH 8.0), 150 mM NaCl, 1 mg/mL DNase I, 1 complete EDTA‐free protease inhibitor tablet (Roche Diagnostics GmbH) for each 50 mL of lysis buffer). Cells were lysed using an EmulsiFlex C5 homogenizer (Avestin, Mannheim, Germany) (3‐4×, 10,000–15,000 psi) and centrifuged. The supernatants were purified using HisTrap HP columns (1 mL, Cytiva Ltd., Little Chalfont, UK) on an AKTA PURE protein purification system (Cytiva Ltd.) with Buffer A (50 mM sodium phosphate (pH 8.0), 150 mM NaCl, and a linear gradient (0%–80% Buffer A + 0.5 M imidazole), 15 column volumes (CV)). Purification buffers for the Cys‐variants contained 0.5 mM TCEP. Protein purity was confirmed using SDS‐PAGE and electrospray ionization mass spectrometry performed on a Xevo G2 mass spectrometer with data analyzed using MassLynx software (Waters UK) (Yusuf Hamied Department of Chemistry, University of Cambridge, UK). The amino acid sequences and masses of the proteins are listed in Table S1.

### 4.2 Alexa Fluor™ 647‐maleimide labeling of Cys‐CTPR variants

Cys‐variants of the CTPR proteins (100 μM, 50 mM sodium phosphate buffer, 150 mM NaCl, pH 8, 0.5 mM TCEP) were labeled by reacting with a 2.5× molar excess of Alexa Fluor™ 647 C_2_ maleimide or Alexa Fluor™ 594 C_2_ maleimide (Invitrogen) and incubated (2 h, room temperature (RT)). One millimolar DTT was added, and excess dye was removed using Pierce dye removal columns as described in the manufacturer's protocol (Thermofisher). UV–vis spectra for the fluorophore‐labeled protein were collected on a Nanodrop 2000 spectrophotometer (Thermofisher) and the protein concentration and labeling efficiency were calculated using Equations (1) and (2) (Alexa Fluor™ 594) and Equations (3) and (4) (Alexa Fluor™ 647):

(1) $$\text{protein concentration}\left(M\right)=\left(A_{280}-\left(A_{590}\times 0.56\right)\right)/\left[\varepsilon_{280}\;\text{of protein}\right]$$

(2) $$\text{labeling efficiency}\left(\%\right)=\left(100\times A_{590}\right)/\left(73,000\times \text{protein concentration}\left(M\right)\right)$$

(3) $$\text{protein concentration}\left(M\right)=\left(A_{280}-\left(A_{650}\times 0.03\right)\right)/\left[\varepsilon_{280}\;\text{of protein}\right]$$

(4) $$\text{labeling efficiency}\left(\%\right)=\left(100\times A_{650}\right)/\left(239,000\times \text{protein concentration}\left(M\right)\right)$$

### 4.3 Circular dichroism spectroscopy

Circular dichroism (CD) measurements were conducted with a Chirascan CD spectrometer (Applied Photophysics, Leatherhead, UK) in 1 mm pathlength Precision Cells (110‐QS; Hellma Analytics, Müllheim, Germany). Protein samples at 5 μM in sodium phosphate buffer were measured across 205–250 nm wavelengths. Spectra, taken at 1 nm intervals every 0.5 s, were recorded at 20°C, then at 90°C, and again at 20°C. Each protein measurement was independently repeated three times, with data baseline corrected for buffer and averaged.

### 4.4 Guanidine hydrochloride denaturation assay

The different guanidine hydrochloride (GdnHCl) concentrations were prepared by mixing the appropriate volumes of 50 mM sodium phosphate buffer pH 7.5, 150 mM NaCl, 7 M GdnHCl, and 50 mM sodium phosphate buffer using a Microlab ML510B (Hamilton, Bonaduz, Switzerland). The protein concentration used was 15 μM and protein and solutions were dispensed into 96‐well, half‐area, black polystyrene plates (Corning) and covered with 96‐well microplate aluminium sealing tape (Corning). Samples were equilibrated at 25°C for 2 h. Emission at 360 nm was measured using a CLARIOstar® Microplate Reader (BMG Labtech, Aylesbury, UK). The top optic was used in precise mode for 2 cycles, with an excitation wavelength of 295 nm and a dichroic PL325nm filter at 25°C. Each measurement was performed in triplicate, and three independent experiments were carried out for each protein variant unless otherwise specified. The data were fit to a two‐state model, with denaturation curves fitted directly in GraphPad Prism 10 as described by Perez‐Riba et al. (Perez‐Riba & Itzhaki, 2017).

### 4.5 α‐Synuclein fibril preparation

A T7‐7 plasmid encoding α‐synuclein (Hoyer et al., 2002) was transformed and expressed in BL21(DE3) *E. coli* (NEB (UK) Ltd.), and purification was carried out as detailed in Bell et al. (2023). α‐Synuclein fibrils were generated by incubating 100 μM α‐synuclein (PBS with 0.01% azide) for 72–96 h (37°C, 200 rpm). After incubation, fibril samples were mixed with Thioflavin‐T (ThT) (250 μM) and ThT fluorescence was measured using a CLARIOstar® Microplate Reader (BMG Labtech) with excitation at 440 nm and emission spectra collected between 465 and 580 nm. The fibrils were centrifuged (10 min, 15,000 g) and the supernatant was removed. The fibril pellets were washed with PBS (2×) followed by centrifugation. The washed fibrils were resuspended in PBS and sonicated (low power, 50% pulse, 15 s) with a Sonopuls probe sonicator (Bandelin, HD 2070) to produce preformed fibrils (PFFs). These PFFs were used to seed fresh α‐synuclein fibrils by adding 10% v/v to monomeric protein and incubating for 24–48 h (37°C, quiescent). All fibril samples were imaged by transmission electron microscopy (TEM) prior to use. For TEM analysis, fibrils were diluted to 5–10 μM and incubated (3 min) on carbon‐coated copper grids (EM Resolutions, Keele, UK), followed by washing with deionized water and staining with UranyLess (Labtech, Rotheram, UK) for 2 min (2×) then washed quickly with water. TEM images were taken on a Tecnai G2 80–200 kV transmission electron microscope (Cambridge Advanced Imaging Centre (CAIC), University of Cambridge). Images were analyzed using the SIS Megaview II Image Capture system.

### 4.6 Dot blot assay

Onto nitrocellulose membranes, 5 μL drops of PBS only, α‐synuclein monomer (10 μM) and α‐synuclein fibrils (10 μM) were applied and dried. This was repeated four times. The membrane was incubated in 2% w/v bovine serum albumin (BSA) in TBS‐T (tris buffered saline with 0.1% v/v Tween 20) (4°C, overnight, roller). The membrane was incubated with a fluorophore‐labeled CTPR variant solution (1 μM, 50 mM phosphate pH 8, 150 mM NaCl) (1 h, RT, roller). The membrane was washed by incubating in TBS‐T (3×, 10 min) and then imaged with a LiCor Odyssey (Li‐Cor, Lincoln, USA) at 600 nm with a 2 min exposure (Alexa Fluor™ 594) or 700 nm with a 2 min exposure (Alexa Fluor™ 647) and analyzed with Empiria Studio™ software (Li‐Cor).

### 4.7 Fibril pulldown assay

Aliquots of α‐synuclein fibrils (35 μL of 70 μM solutions (based on monomer concentration)) were incubated with Alexa Fluor™ 647‐labeled CTPR variants (35 μL of 20 μM solutions) and incubated (30 min, RT). The samples were centrifuged (10 min, 15,000g, RT) and the supernatant was removed. The fibril pellets were washed twice with PBS‐T (phosphate buffered saline with 0.1% v/v Tween20). The pellets were resuspended in 35 μL PBS, and the fluorescence signal (bound to the fibrils) was measured in a 384‐well black microtitre plate (Corning) with a CLARIOstar® Microplate Reader (BMG Labtech, Aylesbury, UK) with an excitation wavelength of 622 nm and collecting the emission spectrum between 650 and 710 nm. The spectra were corrected for the labeling efficiency of each CTPR variant and normalized to the 2XFibSyn‐CTPR4 readings. Data from two independent experiments were averaged.

### 4.8 Microfluidic diffusional sizing experiments

Fabrication and operation of the microfluidic chips for microfluidic diffusional sizing (MDS) experiments have been shown previously (Arosio et al., 2016; McDonald & Whitesides, 2002; Qin et al., 2010). In brief, microfluidic devices were obtained by standard soft‐lithography in polydimethylsiloxane (PDMS, Momentive RTV615, Techsil, Bidford on Avon, UK). Carbon nanoparticles (ca. 13 nm, Plasmachem, Berlin, Germany) were mixed into the PDMS before curing to avoid channel cross‐talk, as previously reported (Herling et al., 2016). After curing, the PDMS slips were bonded onto microscopy slides (Epredia Cut Microscope Slide, ThermoFisher) by application of an oxygen plasma. Sample and co‐flow buffer were loaded onto the chip from reservoirs at the respective inlets by applying a negative pressure at the outlet by means of a glass syringe (Hamilton) connected to a syringe pump (neMESYS, Cetoni GmbH, Korbussen, Germany) with a typical flow rate of 100 μL/min. A custom‐built, inverted epifluorescence microscope was used for the detection of the microfluidic profiles. For excitation, light from a brightfield LED light source (Thorlabs, Newton, NJ, USA) was directed through the Cy5‐4040C‐000 Filter set from Semrock (Laser 2000, Huntingdon, UK) and detected with a charge‐coupled‐device camera (Prime 95B, Photometrics, Tucson, AZ, USA). Images were taken using Micro Manager (Version l.4.23 20170327). Synuclein fibril concentrations are determined by incubation of the fibrils with 4 M GdnHCl and subsequent UV absorption spectroscopy (*ε*_275nm_ = 5600 cm^−1^ M^−1^), and are reported with respect to monomer equivalents. Affinities were determined by determining the hydrodynamic radius of the protein complex between the CTPR variant and α‐Synuclein fibrils at different concentrations of both Alexa‐647 labeled CTPR variant and unlabeled αS PFFs. With fitting this data with respect to two species, that is, bound and unbound CTPR variant, it becomes possible to determine the size of the complex along with the fraction of bound CTPR variant. The fraction of bound CTPR variant can be determined with respect to Equation (5) (Scheidt et al., 2019, 2021).

(5) $$\frac{\left[\mathrm{\alpha SF}\right]}{{\left[F\right]}_{0}}=\left(\frac{{\left[F\right]}_{0}+{\left[\mathrm{\alpha S}\right]}_{0}+K_{d}}{2}\pm \sqrt{{\left(\frac{{\left[F\right]}_{0}+{\left[\mathrm{\alpha S}\right]}_{0}+K_{d}}{2}\right)}^{2}-{\left[\mathrm{\alpha S}\right]}_{0}{\left[F\right]}_{0}}\right)\frac{1}{{\left[F\right]}_{0}}$$

### 4.9 Fluorescence microscopy

μ‐Slide 8 well high glass bottom plates (Ibidi) were treated by cleaning with 1 M KOH (30–45 min) and rinsed with PBS (2×). To the well, a poly‐L‐lysine solution (0.1%) was added and incubated (45 min) followed by washing with PBS (2×). The fibrils bound to Alexa‐647 labeled CTPR proteins (10–15 μM) were prepared as described above, and these were incubated in a well (30 min), followed by the removal of the solution and washing with PBS (1×). Imaging was done on an inverted Olympus IX‐73 microscope (Olympus Corporation, Japan), using a TIRF oil‐immersion objective (Apochromat 100×, NA 1.49 Oil, Olympus) with an exposure time of 0.01 s, 100 frames. After being filtered through a dichroic filter cube (Cairn OptoSpin) and a wavelength‐specific emission filter (Semrock), images were captured with an electron‐multiplying charge‐coupled device (EMCCD) camera (Andor iXon3 897) and processed using ImageJ.

## AUTHOR CONTRIBUTIONS

**Samuel Bismut:** Investigation; methodology; writing – review and editing; formal analysis. **Matthias M. Schneider:** Investigation; methodology; writing – review and editing; formal analysis. **Masashi Miyasaki:** Investigation; methodology; formal analysis. **Yuqing Feng:** Investigation; methodology; formal analysis. **Ellis J. Wilde:** Investigation; methodology; formal analysis. **M. Dylan Gunawardena:** Investigation; methodology; formal analysis. **Tuomas P. J. Knowles:** Funding acquisition; formal analysis; supervision; resources; investigation. **Gabrielle S. Kaminski Schierle:** Funding acquisition; formal analysis; supervision; resources; investigation. **Laura S. Itzhaki:** Conceptualization; funding acquisition; writing – review and editing; formal analysis; supervision; resources; investigation. **Janet R. Kumita:** Conceptualization; funding acquisition; writing – original draft; methodology; formal analysis; supervision; resources; investigation.

## Supporting information


**TABLE S1:** Protein sequences and mass spectrometry analysis.
**FIGURE S1:** GdnHCl‐induced denaturation of CTPR proteins. Purification of CTPR variants yielded samples with concentrations between 150 and 400 μM. (a) FibSyn‐CTPR3, (b) FibSyn2‐CTPR3, and (c) 2XFibSyn‐CTPR4CTPR4. 1 μM of protein in 50 mM sodium phosphate, 150 mM NaCl, pH 7.5 and increasing GdnHCl concentrations (from 0 to 6.4 M) at 25°C. Excitation was at 295 nm and emission intensity was measured at 360 nm. A minimum of three independent experiments were recorded for each protein. The data were fitted with a two‐state model to give the midpoint of unfolding and m value (a constant proportional to the change in solvent‐accessible surface area upon unfolding). Error bars show the standard error of the mean.
**FIGURE S2:** CD analysis and GdnHCl‐induced denaturation of CTPR3 and CTPR4 (negative controls). To determine whether fibril‐binding is due to the presence of the peptide motifs, CTPR3 and CTPR4 containing the native inter‐repeat loops were used. CD analysis of (a) CTPR3 and (b) CTPR4 was performed with 5 μM of protein in 50 mM sodium phosphate, 150 mM NaCl, 0.5 mM TCEP, pH 7.5, spectra recorded from 205 to 250 nm in 0.5 nm intervals. Samples were measured at 20°C (black), 90°C (red) and return to 20°C (blue). GdnHCl denaturation was performed for (c) CTPR3 and (d) CTPR4 with 1 μM of protein in 50 mM sodium phosphate, 150 mM NaCl, pH 7.5 and increasing GdnHCl concentration (from 0 to 6.4 M) at 25°C. Excitation was at 295 nm and emission intensity was measured at 360 nm. A minimum of three independent experiments were recorded for each protein. The data were fitted to a two‐state model to give the midpoint of unfolding and *m* value (a constant proportional to the change in solvent‐accessible surface area upon unfolding). Error bars show the standard error of the mean.
**FIGURE S3:** Dot blot assay to test CTPR variant binding to monomeric and fibrillar α‐synuclein. Monomeric and fibrillar α‐synuclein samples were applied to nitrocellulose membranes followed by incubation with (a) Alexa Fluor™ 594‐labeled CTPR variants and (b) Alexa Fluor™ 647‐labeled CTPR variants. Imaging using a LiCor Odessey system shows no interactions between the fluorophore‐labeled CTPRs and monomeric α‐synuclein and increased interactions between fibrillar α‐synuclein and CTPR variants containing the fibril binding peptide motifs. Some non‐specific binding was observed with the Alexa Fluor™ 647‐labeled CTPR3 negative control (also observed in the fibril‐pulldown assay), this non‐specific binding was not observed with the Alexa Fluor™ 594‐labeled CTPR3 negative control.
**FIGURE S4:** Comparing CTPR variants interactions with α‐synuclein fibrils versus Aβ1–42 fibrils. (a) Fluorescence emission spectrum for CTPR3 (negative control) and FibSyn‐CTPR3 bound α‐synuclein fibrils showing specific interactions. (b) Fluorescence emission spectrum for CTPR3 (negative control) and FibSyn‐CTPR3 bound Aβ1–42 fibrils, showing no specific interactions with FibSyn‐CTPR3. (c) Representative TEM image of Aβ1–42 fibrils used for fibril pull‐down and MDS assays. (d) MDS assays of Alexa Fluor™ 647‐labeled CTPR variants with increasing concentrations of Aβ1–42 fibrils. No change in hydrodynamic radius is seen for any of the FibSyn‐CTPRs.

## Data Availability

Data sharing is not applicable to this article as no new data were created or analyzed in this study.
