# Supplementary material for: Using a stable protein scaffold to display peptides that bind to alpha‐synuclein fibrils
Source: Protein Sci. 2025 May 15;34(6):e70150. doi: 10.1002/pro.70150 (PMC12079352; doi:10.1002/pro.70150)
Supplement: Supplementary file 1 — TABLE S1: Protein sequences and mass spectrometry analysis. FIGURE S1: GdnHCl‐induced denaturation of CTPR proteins. Purification of CTPR variants yielded samples with concentrations between 150 and 400 μM. (a) FibSyn‐CTPR3, (b) FibSyn2‐CTPR3, and (c) 2XFibSyn‐CTPR4CTPR4. 1 μM of protein in 50 mM sodium phosphate, 150 mM NaCl, pH 7.5 and increasing GdnHCl concentrations (from 0 to 6.4 M) at 25°C. Excitation was at 295 nm and emission intensity was measured at 360 nm. A minimum of three independent experiments were recorded for each protein. The data were fitted with a two‐state model to give the midpoint of unfolding and m value (a constant proportional to the change in solvent‐accessible surface area upon unfolding). Error bars show the standard error of the mean. FIGURE S2: CD analysis and GdnHCl‐induced denaturation of CTPR3 and CTPR4 (negative controls). To determine whether fibril‐binding is due to the presence of the peptide motifs, CTPR3 and CTPR4 containing the native inter‐repeat loops were used. CD analysis of (a) CTPR3 and (b) CTPR4 was performed with 5 μM of protein in 50 mM sodium phosphate, 150 mM NaCl, 0.5 mM TCEP, pH 7.5, spectra recorded from 205 to 250 nm in 0.5 nm intervals. Samples were measured at 20°C (black), 90°C (red) and return to 20°C (blue). GdnHCl denaturation was performed for (c) CTPR3 and (d) CTPR4 with 1 μM of protein in 50 mM sodium phosphate, 150 mM NaCl, pH 7.5 and increasing GdnHCl concentration (from 0 to 6.4 M) at 25°C. Excitation was at 295 nm and emission intensity was measured at 360 nm. A minimum of three independent experiments were recorded for each protein. The data were fitted to a two‐state model to give the midpoint of unfolding and m value (a constant proportional to the change in solvent‐accessible surface area upon unfolding). Error bars show the standard error of the mean. FIGURE S3: Dot blot assay to test CTPR variant binding to monomeric and fibrillar α‐synuclein. Monomeric and fibrillar α‐synuclein samples were [file PRO-34-e70150-s001.pdf]

Supplementary Information

Using a stable protein scaffold to display peptides that bind to alpha-synuclein fibrils

Samuel Bismut[1,4], Matthias M. Schneider[2,4], Masashi Miyasaki[1], Yuqing Feng[3], Ellis J. Wilde[1], M. Dylan Gunawardena[1], Tuomas P.J. Knowles[2], Gabrielle S. Kaminski Schierle[3], Laura S. Itzhaki[1] and Janet R. Kumita[1,*]

[1]Department of Pharmacology, University of Cambridge, Tennis Court Road, Cambridge, UK, CB2 1PD

[2]Yusuf Hamied Department of Chemistry, University of Cambridge, Lensfield Road, Cambridge, UK, CB2 1EW

[3]Department of Chemical Engineering and Biotechnology, University of Cambridge, Cambridge, UK, CB3 0AS

[4]These authors contributed equally

*To whom correspondence should be addressed: jrk38@cam.ac.uk

Table S1: Protein sequences and mass spectrometry analysis

Amino acid sequences for the different CTPR variants show a solvating helix (blue) and an N-terminal His-tag at the N-terminus (italics). Fibril-binding motifs are shown in bold/italics and they are flanked by DPNN linker regions (underlined).

Protein	Amino acid sequence	Expected Mass (Da)	Observed Mass (Da)
CPTR3	MRGS*HHHHHH*GLVPRGSAEAWYNLGNAYYKQGDYQKAIEYYQKALELDPRSAEAWYNLGNAYYKQGDYQKAIEYYQKALELDPRSAEAWYNLGNAYYKQGDYQKAIEYYQKALELDPRSAEAKQNLGNAKQKQG	15633.2	15633.2 ± 1.0
Cys-CTPR3	MRGS*HHHHHH*GLVPRGCAEAWYNLGNAYYKQGDYQKAIEYYQKALELDPRSAEAWYNLGNAYYKQGDYQKAIEYYQKALELDPRSAEAWYNLGNAYYKQGDYQKAIEYYQKALELDPRSAEAKQNLGNAKQKQG	15649.2	15648.6 ± 0.2
FibSyn-CTPR3	MRGS*HHHHHH*GLVPRGSAEAWYNLGNAYYKQGDYQKAIEYYQKALELDPNN***KLVFFAE***DPNNAEAWYNLGNAYYKQGDYQKAIEYYQKALELDPRSAEAWYNLGNAYYKQGDYQKAIEYYQKALELDPRSAEAKQNLGNAKQKQG	16893.6	16892.6 ± 0.7
Cys-FibSyn-CTPR3	MRGS*HHHHHH*GLVPRGCAEAWYNLGNAYYKQGDYQKAIEYYQKALELDPNN***KLVFFAE***DPNNAEAWYNLGNAYYKQGDYQKAIEYYQKALELDPRSAEAWYNLGNAYYKQGDYQKAIEYYQKALELDPRSAEAKQNLGNAKQKQG	16909.6	16915.3 ± 6.0
FibSyn2-CTPR3	MRGS*HHHHHH*GLVPRGSAEAWYNLGNAYYKQGDYQKAIEYYQKALELDPNN***KLVFWAK***DPNNAEAWYNLGNAYYKQGDYQKAIEYYQKALELDPRSAEAWYNLGNAYYKQGDYQKAIEYYQKALELDPRSAEAKQNLGNAKQKQG	16931.6	16931.7 ± 0.1
Cys-FibSyn2-CTPR3	MRGS*HHHHHH*GLVPRGCAEAWYNLGNAYYKQGDYQKAIEYYQKALELDPNN***KLVFWAK***DPNNAEAWYNLGNAYYKQGDYQKAIEYYQKALELDPRSAEAWYNLGNAYYKQGDYQKAIEYYQKALELDPRSAEAKQNLGNAKQKQG	16947.7	16947.7 ± 0.2
CTPR4	MRGS*HHHHHH*GLVPRGSAEAWYNLGNAYYKQGDYQKAIEYYQKALELDPNNAEAWYNLGNAYYKQGDYQKAIEYYQKALELDPNNAEAWYNLGNAYYKQGDYQKAIEYYQKALELDPNNAEAWYNLGNAYYKQGDYQKAIEYYQKALELDPNN	18048.6	18054.8 ± 6.7
Cys-CTPR4	MRGS*HHHHHH*GLVPRGCAEAWYNLGNAYYKQGDYQKAIEYYQKALELDPNNAEAWYNLGNAYYKQGDYQKAIEYYQKALELDPNNAEAWYNLGNAYYKQGDYQKAIEYYQKALELDPNNA	18064.7	18066.5 ± 2.8

	EAWYNLGNAYYKQGDYQKAIEYYQKALELDPNN		
2XFibSyn-CTPR4	MRGSHHHHHHGLVPRGSAEAWYNLGNAYYKQGDYQKAIEYYQKALELDPNN***KLVFFAE***DPNNAEAWYNLGNAYYKQGDYQKAIEYYQKALELDPNNAEAWYNLGNAYYKQGDYQKAIEYYQKALELDPNN***KLVFFAE***DPNNAEAWYNLGNAYYKQGDYQKAIEYYQKALELDPNN	20599.5	20599.0 ± 2.0
Cys-2XFibSyn-CTPR4	MRGSHHHHHHGLVPRGCAEAWYNLGNAYYKQGDYQKAIEYYQKALELDPNN***KLVFFAE***DPNNAEAWYNLGNAYYKQGDYQKAIEYYQKALELDPNNAEAWYNLGNAYYKQGDYQKAIEYYQKALELDPNN***KLVFFAE***DPNNAEAWYNLGNAYYKQGDYQKAIEYYQKALELDPNN	20615.6	20615.6 ± 0.2

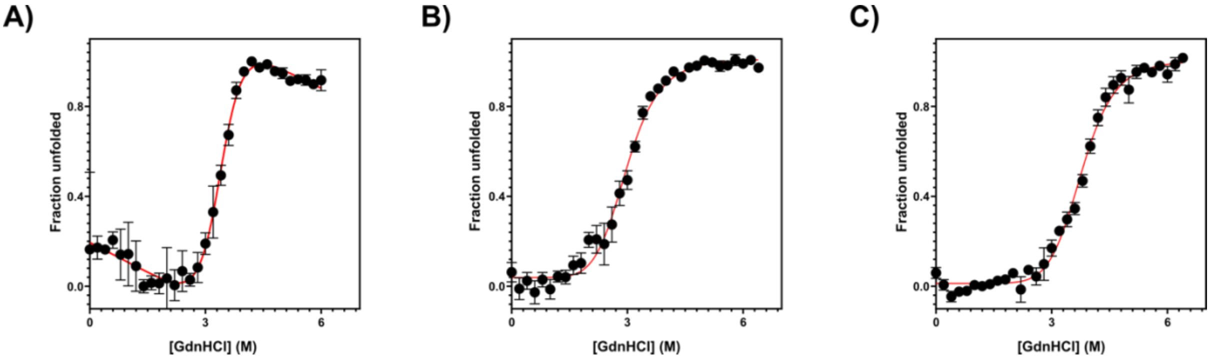

Fig. S1. GdnHCl-induced denaturation of CTPR proteins. Purification of CTPR variants yielded samples with concentrations between 150-400 μM. A) FibSyn-CTPR3, B) FibSyn2-CTPR3 and C) 2XFibSyn-CTPR4CTPR4. 1 μM of protein in 50 mM sodium phosphate, 150 mM NaCl, pH 7.5 and increasing GdnHCl concentrations (from 0 M to 6.4 M) at 25°C. Excitation was at 295 nm and emission intensity was measured at 360 nm. A minimum of three independent experiments were recorded for each protein. The data were fitted with a two-state model to give the midpoint of unfolding and *m* value (a constant proportional to the change in solvent-accessible surface area upon unfolding). Error bars show the standard error of the mean.

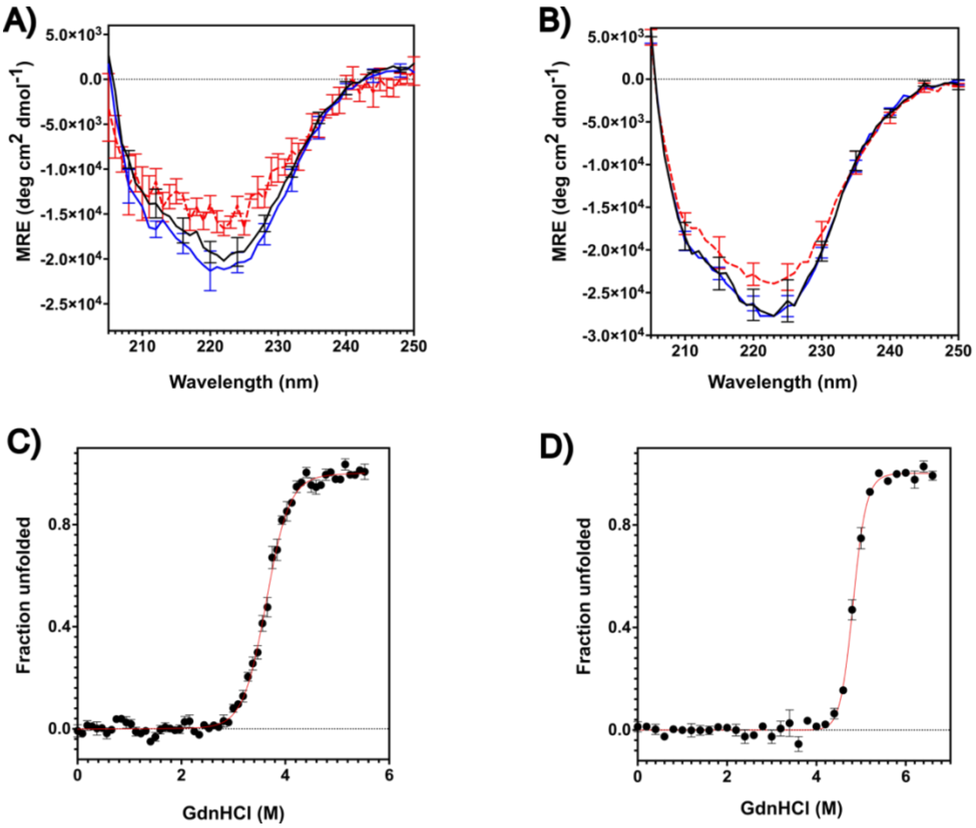

Fig. S2. CD analysis and GdnHCl-induced denaturation of CTPR3 and CTPR4 (negative controls). To determine whether fibril-binding is due to the presence of the peptide motifs, CTPR3 and CTPR4 containing the native inter-repeat loops were used. CD analysis of A) CTPR3 and B) CTPR4 was performed with 5 µM of protein in 50 mM sodium phosphate, 150 mM NaCl, 0.5 mM TCEP, pH 7.5, spectra recorded from 205 nm to 250 nm in 0.5 nm intervals. Samples were measured at 20°C (black), 90°C (red) and return to 20°C (blue). GdnHCl denaturation was performed for C) CTPR3 and D) CTPR4 with 1 µM of protein in 50 mM sodium phosphate, 150 mM NaCl, pH 7.5 and increasing GdnHCl concentration (from 0 M to 6.4 M) at 25°C. Excitation was at 295 nm and emission intensity was measured at 360 nm. A minimum of three independent experiments were recorded for each protein. The data were fitted to a two-state model to give the midpoint of unfolding and *m* value (a constant proportional to the change in solvent-accessible surface area upon unfolding). Error bars show the standard error of the mean

A)

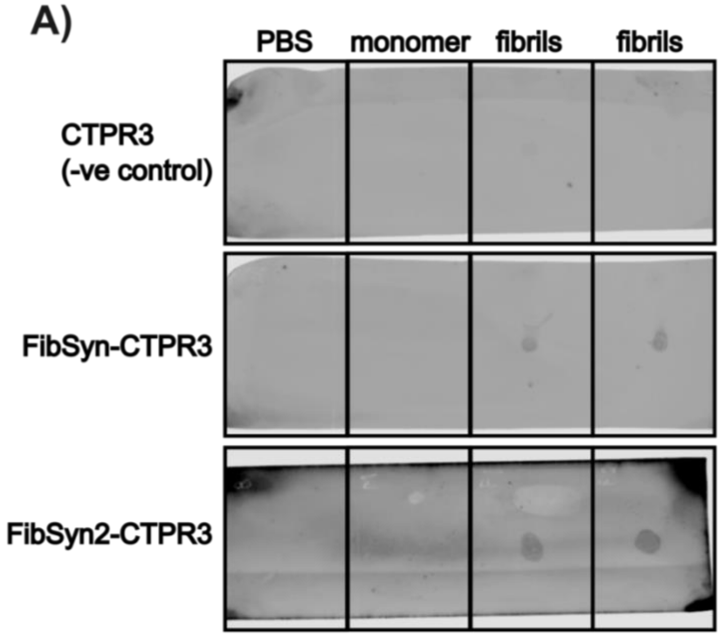

B)

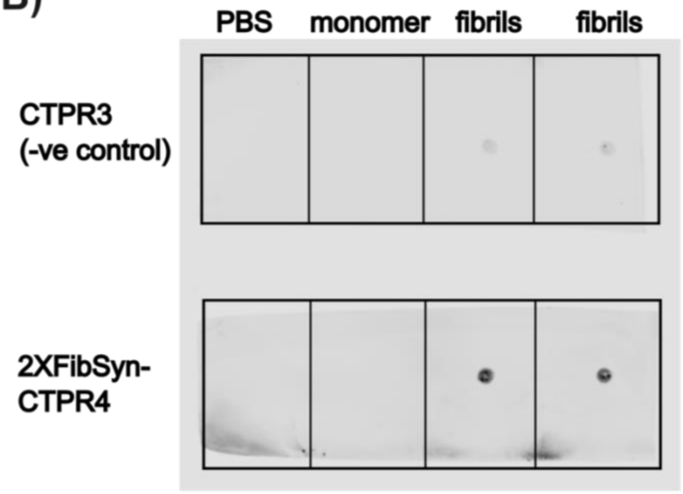

Fig. S3. Dot blot assay to test CTPR variant binding to monomeric and fibrillar α-synuclein. Monomeric and fibrillar α-synuclein samples were applied to nitrocellulose membranes followed by incubation with A) Alexa Fluor™ 594-labelled CTPR variants and B) Alexa Fluor™ 647-labelled CTPR variants. Imaging using a LiCor Odessey system shows no interactions between the fluorophore-labelled CTPRs and monomeric α-synuclein and increased interactions between fibrillar α-synuclein and CTPR variants containing the fibril-binding peptide motifs. Some non-specific binding was observed with the Alexa Fluor™ 647-labelled CTPR3 negative control (also observed in the fibril-pulldown assay), this non-specific binding was not observed with the Alexa Fluor™ 594-labelled CTPR3 negative control.

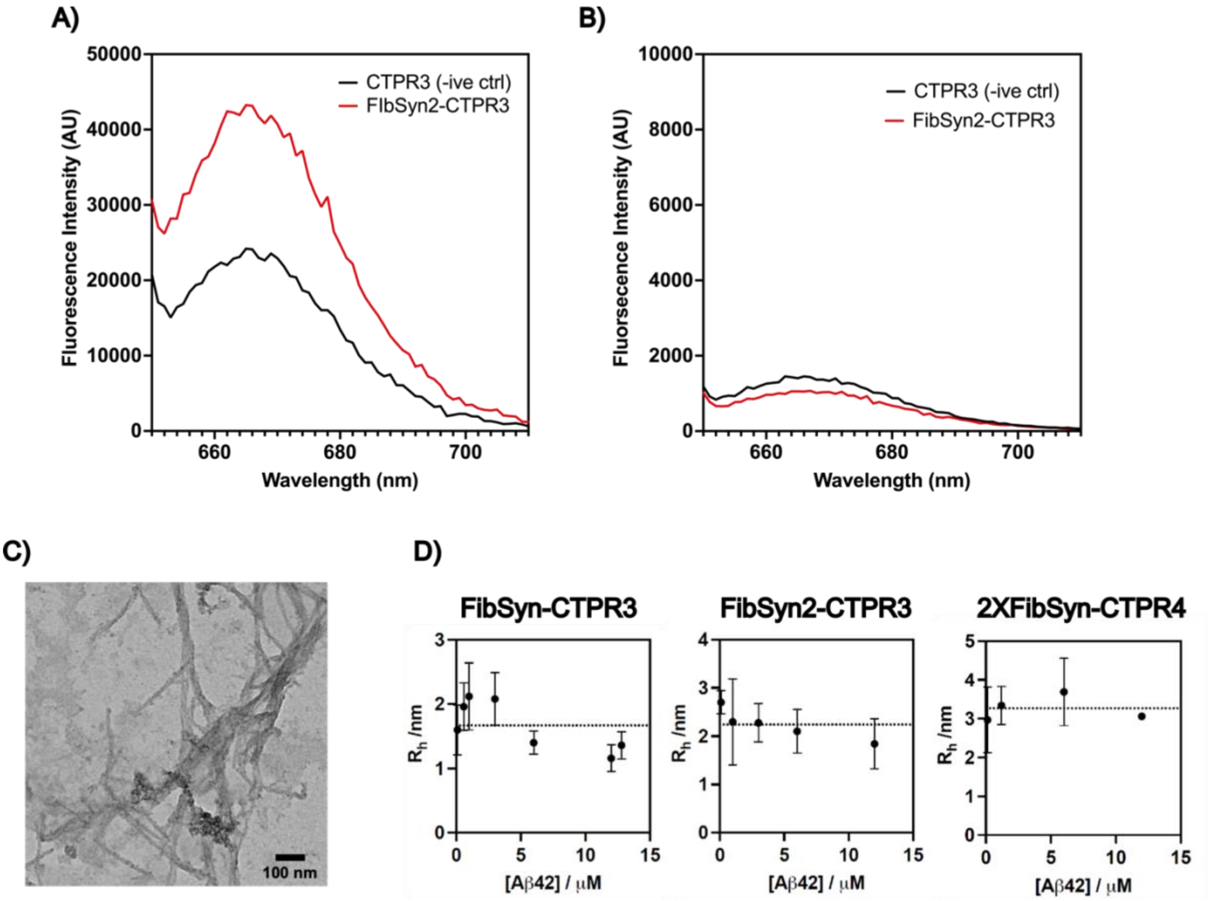

Fig S4. Comparing CTPR variants interactions with α-synuclein fibrils versus A$\beta_{1\text{-}42}$ fibrils. A) Fluorescence emission spectrum for CTPR3 (negative control) and FibSyn-CTPR3 bound α-synuclein fibrils showing specific interactions. B) Fluorescence emission spectrum for CTPR3 (negative control) and FibSyn-CTPR3 bound A$\beta_{1\text{-}42}$ fibrils, showing no specific interactions with FibSyn-CTPR3. C) Representative TEM image of A$\beta_{1\text{-}42}$ fibrils used for fibril pull-down and MDS assays. D) MDS assays of Alexa Fluor^{TM} 647-labelled CTPR variants with increasing concentrations of A$\beta_{1\text{-}42}$ fibrils. No change in hydrodynamic radius is seen for any of the FibSyn-CTPRs.
